# Vitamin D status and body composition: a cross-sectional study among employees at a private university in Lebanon

**DOI:** 10.1186/s40795-018-0239-6

**Published:** 2018-07-26

**Authors:** Sibelle Al Hayek, Jocelyne Matar Bou Mosleh, Rachelle Ghadieh, Jessy El Hayek Fares

**Affiliations:** 1 Department of Dietetics and Nutrition, The University of Kansas Medical Center, 3901 Rainbow Blvd, Kansas City, KS 66160 USA; 2 Department of Nursing and Health Sciences, Notre Dame University- Louaize (NDU), Zouk Mosbeh, Lebanon; 3 Department of Endocrinology, Diabetes, Metabolism and Eating Disorders, University Hospital of Saint-Etienne, Saint-Etienne Cedex, France

**Keywords:** Vitamin D status, Vitamin D intake, Percent body fat, Lebanese, Body composition, Adults

## Abstract

**Background:**

The prevalence of low vitamin D status is increasing globally, and Lebanon is not spared. The objectives of this study are to determine the prevalence and correlates of low vitamin D status, and to assess the association between percent body fat and vitamin D status, independently of obesity.

**Methods:**

A cross-sectional study was performed on NDU employees. Data on dietary intake, physical activity, lifestyle, health status, and demographic variables were collected during a face-to-face interview. Anthropometric measures (weight, height and waist circumference) were measured and body composition was assessed using the bioelectrical impedance analysis (BIA) machine InBody 720 (Biospace, Seoul, Korea). The Nutritionist Pro diet analysis software version 31.0 was used to estimate dietary intake of vitamin D. Serum 25 hydroxyvitamin D (25(OH)D) was measured using enzyme linked immunosorbent assay kit (ELISA) (Calbiotech, Spring Valley, California, USA). Vitamin D status was assessed according to the National Osteoporosis Foundation (sufficiency: ≥ 75 nmol/L / ≥30 ng/mL) and the Institute of Medicine cut-offs (adequacy: ≥50 nmol/L / ≥20 ng/mL). Statistical analyses were performed by SPSS version 22.

**Results:**

A total of 344 employees (50% Male) aged between 20 and 74 years participated in the study. More than half of the participants were overweight and obese. Mean serum vitamin D concentrations were 28.2 ± 13.9 ng/mL. Among participants, 37.5% of our study population had 25(OH)D ≥ 30 ng/mL, and 68.3% had 25(OH)D ≥ 20 ng/mL. Individuals with low vitamin D status had significantly higher percent body fat (PBF) (*p* < 0.005), and higher waist circumference (WC) (*p* = 0.012) than in the sufficient group, however BMI did not differ by vitamin D status. Logistic regression analysis indicated that a 1% increase in body fat increases the odds of having 25(OH)D ≤ 30 ng/mL by 8% while controlling for BMI and other confounders (*p* = 0.019).

**Conclusion:**

This study reinforces the need for regular screening for low vitamin D status in Lebanese adults, particularly individuals at risk, including those with high risk WC, high PBF, who work indoors and have low vitamin D intake, and recommending vitamin D supplementation if needed.

## Background

Vitamin D is a fat soluble compound that can be obtained from two sources: primarily sunlight exposure and diet (e.g: fatty fish, egg yolk, cod-liver, fortified foods, and supplements) [1]. Vitamin D refers to vitamin D_2_ (ergocalciferol) or vitamin D_3_ (cholecalciferol) [1].

Measurement of serum 25 hydroxyvitamin D (25(OH)D) concentration is the best indicator to assess vitamin D status, since it indicates both endogenous production and dietary vitamin D intake. The Institute of Medicine (IOM) recommends concentrations of 25(OH)D to be ≥20 ng/mL (≥50 nmol/L) [2], while other institutes, such as the National Osteoporosis Foundation (NOF) and the American Geriatrics Society (AGS) recommend concentrations of 25(OH)D to be > 30 ng/mL (> 75 nmol/L) [3]. The inconsistency in the definition of healthy vitamin D status depends mainly on the health outcome considered.

The prevalence of low vitamin D status is increasing globally. Levels of 25(OH)D < 10 ng/mL in adults were most common in Asia (78%), compared to Europe (2–30%), and North America (13%) [4]. Despite the sunny weather in Lebanon, multiple studies have reported a high prevalence of low vitamin D status in the Lebanese population. For instance, Hoteit et al., 2014 reported that 60% of Lebanese adults had 25(OH)D <  20 ng/mL [5], while Rachkidi and Aoun, 2015 reported a higher prevalence of low vitamin D status among ambulatory patients (73.33%), using a higher cutoff (25(OH)D < 30 ng/mL), [6].

Low serum vitamin D concentrations have been reported among obese individuals [7]. The high prevalence of low vitamin D status paralleled the pandemic of obesity [8]. Both conditions, obesity and low vitamin D status, have been implicated in the development of many chronic diseases including type 2 diabetes, cancer, and cardiovascular diseases [9]. Accordingly, more research is focusing on the understanding of the relationship between vitamin D status and obesity [8]. Studies in the Middle East assessing this relationship are limited [10–12], even though many countries in the Middle East reported high prevalence rates of low vitamin D status and obesity [5, 9, 13]. Gannage et al., 2010 showed that 25(OH)D was inversely correlated with BMI (*r* = − 0.18; *p* < 0.01) and WC (*r* = − 0.19; p < 0.01) among 381 Lebanese non-obese university students. However, these results do not necessarily apply to overweight and obese individuals in older age groups, who are at higher risk of chronic disease [14]. Even though the association between serum 25(OH)D levels and percent body fat (PBF) has been explored in the literature, this association has only been explored in the Arab region in Emirati obese diabetic adults. One cross-sectional study among 309 obese diabetic adults (age range: 30–60 years) in UAE showed that the prevalence of 25(OH)D < 50 nmol/L was 83.2%. Furthermore, serum 25(OH)D correlated negatively with body mass index (BMI) (*r* = − 0.15, *p* < 0.05), waist circumference (WC) (*r* = − 0.17, p < 0.05), and fat mass (*r* = − 0.16, p < 0.05) [10]. The results of this study cannot be generalized to the general population, as the study participants were diabetic and obese, and the relationship between vitamin D status and obesity could be altered by diabetes. In other regions across the world, the results of studies on the association between serum 25(OH)D levels and PBF were inconsistent. This controversy in the literature could be due to the fact that the correlates and confounders (vitamin D intake, sun exposure, sunscreen use, age, etc.) affecting the relationship between vitamin D status and percentage body fat (PBF) were not assessed thoroughly. Further, the association between obesity and vitamin D status could also differ between gender and among different ethnic groups [15–17]. There is a substantial body of evidence supporting a genetic basis for low 25(OH)D concentrations in the Middle East. It has been shown previously that people from the Middle Eastern region have a specific phenotype that exposes them to low vitamin D status [18]. Therefore, the independent association between vitamin D status and PBF still needs further examination.

Accordingly, our objectives were to examine the association of socio-demographic, lifestyle, dietary, and anthropometric factors with vitamin D status, and to assess the independent association between PBF and vitamin D status among university employees in Lebanon. We hypothesized that people with higher total PBF will have lower serum vitamin D concentrations, independent of BMI.

To our knowledge, this study is the first to assess the association between serum 25(OH)D concentrations and PBF in Lebanon.

## Methods

### Study design and recruitment methods

A cross-sectional study was carried out on Notre Dame University (NDU) employees, in the Zouk Mosbeh, North, and Shouf campuses. Prior to the initiation of the study, the study protocol was approved by the Institutional Review Board of NDU.

Beginning in October 2016, an e-invite was sent to all staff and faculty members of NDU to invite them to participate in the study. Following the e-invite, four nutritionists visited all faculty and staff members in their offices to encourage participation. Of the 600 contacted employees in the three NDU campuses, 360 accepted to participate and were screened for eligibility. Exclusion criteria included pregnancy, lactation, failure to complete the questionnaires, and presence of a pacemaker or metal pieces in the participant’s body. Those who were found to be eligible (*n* = 344) were asked to sign an informed consent form and then contacted by the study investigators to arrange for a 30-min face-to-face interview. An identification number was assigned to each participant. All questionnaires were labeled using codes. The investigators maintained the list associating names with codes and were in charge of keeping it confidential.

### Data collection procedures

During the 30-min face-to-face interview, trained nutritionists filled out three questionnaires (background questionnaire, short-form of the International Physical Activity Questionnaire [IPAQ-short form] and food frequency questionnaire [FFQ]). All questionnaires were pre-tested using a sample of thirty NDU employees in the three campuses. Revisions and corrections were performed before initializing the study. The background questionnaire assessed demographic (i.e income, age, gender, marital status, etc.) and lifestyle variables (i.e sun exposure practices, sunscreen use, smoking, alcohol intake, etc.). The IPAQ-short form was used to assess the level of physical activity. IPAQ asks about three specific types of activities: walking, moderate and vigorous physical activities and, time spent by an individual [19]. The items were structured to provide separate scores on each of these activities. Using the following values, Walking = 3.3 METs, Moderate PA = 4.0 METs and Vigorous PA = 8.0 METs, four continuous scores were calculated. These scores were then added to calculate the total physical activity score. Low-level, moderate-level and high-level PA were defined by scores of less than 600 MET-minutes per week, between 600 to less than 3000 MET-minutes per week, and of 3000 or more MET-minutes per week, respectively. The FFQ was comprised of 9 food items. For each food item, participants were asked to mark their frequency of intake of a designated serving/portion size per day/week/month or rarely/never during the past year. The FFQ included full-fat/low-fat dairy products, eggs and egg-based dishes, fish, margarine, cheeses, and ice cream. Dietary intake of vitamin D was assessed using an adapted version of an existing prototype food frequency questionnaire specific for assessment of vitamin D intake that was developed by study investigators [20]. The Nutritionist Pro diet analysis software, version 31.0 (Axxya Systems, Woodinville, WA, USA) was used to generate estimates of dietary intake of vitamin D [21–23]. Lebanese dishes and recipes were composed and entered using this software according to the Middle-East Food Composition Tables and the Canadian Nutrient File [23]. At the end of the interview, study participants were invited to the nutrition laboratory to collect anthropometric (height, WC, weight and body composition) and biochemical measurements after an overnight fast. Height was measured to the nearest 0.1 cm according to the following protocol: no shoes, heels together, and head touching the stadiometer’s ruler aligned horizontally. For WC measurement, a non-stretchable tailor measuring tape was placed around the bare abdomen just above the hip bone and parallel to the floor. Participants were asked to exhale, and measurement was taken to the nearest centimeter at the midpoint between the bottom of the rib cage and above the top of the iliac crest during minimal respiration. WC values were classified as high risk/low risk for diseases, using the World Health Organization (WHO) cutoffs for men > 102 cm and women > 88 cm [24]. Weight and body composition were assessed using the bioelectrical impedance analysis (BIA) machine InBody 720 (Biospace, Seoul, Korea). BIA is widely used in research; it is quick, safe, and inexpensive [25]. BIA measures body water, then estimates fat mass and fat-free mass [26]. The BIA machine was transported to different campuses for data collection, and it was calibrated prior to its use. The participants were asked to stand on the machine barefooted, without wearing any metal/jewelry, after wiping hands and feet with electrolyte wipes. BMI was calculated as: Weight (kg)/ Height (m^2^). Underweight was defined as BMI < 18.5 kg/m^2^, normal weight: 18.5–24.9 kg/m^2^, overweight: 25–29.9 kg/m^2^, and obese ≥30 kg/m^2^ [24].

Upon the visit to the nutrition laboratory, a nurse collected a fasting sample of blood. Samples collected at the regional campuses were transported to the Zouk Mosbeh campus on ice. Samples were stored at -20 °C in the freezer for a maximum of 6 weeks before analysis. Serum 25(OH)D concentrations were measured at the Biology laboratory at NDU Zouk Mosbeh campus using enzyme linked immunosorbent assay kit (ELISA) (Calbiotech, Spring Valley, California, USA), with an intra-assay coefficient variation of 4.95% and an inter-assay coefficient variation of 5.63%, and a sensitivity of 0.67 ng/mL.

Since there are no universal cutoff values for categorizing vitamin D status, serum 25(OH)D concentration was compared to different cutoffs, specifically the Institute of Medicine (IOM) and National Osteoporosis Foundation (NOF). According to the IOM, serum 25(OH)D concentrations less than 12 ng/mL (< 30 nmol/L) are associated with vitamin D deficiency, concentrations between 12 to less than 20 ng/mL (30 to < 50 nmol/L) are generally considered inadequate for bone and overall health in healthy individuals, and serum 25(OH)D ≥ 20 ng/mL (≥ 50 nmol/L) concentrations are considered adequate [2]. However, the National Osteoporosis Foundation (NOF) classifies the vitamin D status as deficient if serum 25(OH)D levels are ≤20 ng/mL (≤50 nmol/L), insufficient if levels are > 20–30 ng/mL (> 50–75 nmol/L), and sufficient if levels are > 30 ng/mL (> 75 nmol/L) [3].

### Statistical analyses

Assuming that the prevalence rate of vitamin D deficiency among Lebanese adults was 73% [5], the sample size was calculated to be 303 individuals. Quantitative and qualitative measurements were summarized as mean ± standard deviation and n (%), respectively. Comparisons of continuous and categorical variables were performed using independent sample T Test/Mann-Whitney-U-test and the chi square test /Fisher’s exact test, respectively. Two logistic regression models were used, where low vitamin D status (defined as 25(OH)D ≤ 30 ng/mL or ≤ 20 ng/mL) was used as the dependent variable and PBF was used as the independent variable, controlling for BMI, age, gender, sunlight exposure, vitamin D intake, vitamin D supplements use, alcohol intake, intake of oral contraceptive pills (OCP), education, sunscreen use, chronic illness status, income, physical activity, cholesterol, triglycerides, HDL, and LDL levels. Statistical analyses were performed using the Statistical Package for Social Sciences (SPSS) version 22 for Windows. A *p*-value of less than 0.05 was considered statistically significant.

## Results

A total of 344 subjects (50% men and 50% women) aged between 20 and 74 years participated in the study. Characteristics of the study population stratified by gender are summarized in Table 1. The sample consisted mostly of educated participants holding a university degree (78.2%), married (65.4%), with an income of less than $4000 per month (57.9%). The majority of study participants were non-smokers (61.6%) and did not drink alcohol (74.1%). More than half of the participants (64.2%) had a low level of physical activity. Daily mean vitamin D intake was 2.2 ± 3.2 μg. Of the participants, 21.5% were taking vitamin D supplements. More than one third of the participants were of a healthy weight (35.5%), while the majority of participants were overweight and obese (64.0%). Men were older, had lower PBF, had a higher milk intake, reported higher prevalence of medical morbidities, smoking and drinking, overweight and obesity, spending > 60 mins in the sun, and hypertriglyceridemia compared to women (*p* < 0.005). However, men had a lower prevalence of sunscreen use (4.1%) compared to women (54.1%, p < 0.005).

**Table 1:** Sample Characteristics (Socio-demographic, Dietary, Lifestyle, Anthropometric, and Biochemical factors) of Study Participants

Characteristic	Total (n = 344)		Men (*n* = 172)		Women (n = 172)		*P*-value^4^
	n or mean or median	% or SD or IQR	n or mean	% or SD	n or mean	% or SD	
Age (years)	42.6	11.5	45.6	12.1	39.5	10.2	0.000
Marital status							
Single/ Separated/ Divorced	119	34.6	53	30.8	66	38.4	0.174
Married	225	65.4	119	69.2	106	61.6	
Education level							
High school	75	21.8	48	27.9^a^	27	15.7^b^	0.013
Bachelor degree	87	25.3	36	20.9^a^	51	29.6^a^	
Graduate	182	52.9	88	51.2^a^	94	54.7^a^	
Income ($)							
< 2250	112	32.6	61	35.5^a^	51	29.6^a^	0.033
2250–4000	87	25.3	33	19.2^a^	54	31.4^b^	
> 4000	145	42.2	78	45.3^a^	67	39.0^a^	
Medical morbidity							
No	202	58.9	91	53.2	111	64.5	0.043
Yes	141	41.1	80	46.8	61	35.5	
Vitamin D intake (μg)	1.3	[0.6–3.2]	2.5	4.0	2.0	2.0	0.209
Intake of vitamin D supplement, past 3 months							
No	270	78.5	142	82.6	128	74.4	0.088
Yes	74	21.5	30	17.4	44	25.6	
Alcohol drinking							
No	255	74.1	112	65.1	143	83.1	0.000
Yes	89	25.9	60	34.9	29	16.9	
Smoking							
No	212	61.6	93	54.1	119	69.2	0.006
Yes	132	38.4	79	45.9	53	30.8	
Daily exposure to direct sunlight							0.008
≤ 15 mins	136	39.5	66	38.4^a^	70	40.7^a^	
16–60 min	112	32.6	46	26.7^a^	66	38.4^b^	
> 60 mins	96	27.9	60	34.9^a^	36	20.9^b^	
Use sunscreen							
No	244	70.9	165	95.9	79	45.9	0.000
Yes	100	29.1	7	4.1	93	54.1	
Physical activity level							
Low	221	64.2	10.3	59.9	118	68.6	0.115
Moderate/High	123	35.8	69.0	40.1	54	31.4	
OCP^1^ use							
No	341	99.1	100	100	169	98.3	0.248
Yes	3	0.9	0	0	3	1.7	
BMI^2^							
Underweight	2	0.6	0	0.0^a,b^	2	1.2^a,b^	0.000
Normal	122	35.5	27	15.7^a^	95	55.2^b^	
Overweight	130	37.8	86	50.0^a^	44	25.6^b^	
Obese	90	26.2	59	34.3^a^	31	18.0^b^	
Percent body fat	30.9	7.9	27.8	6.9	34.0	7.6	0.000
Waist circumference							
Low risk^3^	170	49.4	85	49.4	85	49.4	1
High risk	174	50.6	87	50.6	87	50.6	
Vitamin D concentration (ng/mL)	25.7	[18.2–25.7]	28.4	15.0	28.1	12.8	0.846

^1^Oral Contraceptive Pills

^2^Body Mass Index

^3^WC values were classified as high risk/low risk for diseases, using the World Health Organization (WHO) cutoffs for men > 102 cm and women > 88 cm [24]

^4^The *P* value reflects gender differences

Columns with superscripts without a common symbol differ, the P value is < 0.05

Comparisons of continuous and categorical variables were performed using independent sample T Test/Mann-Whitney-U-test and the chi square test /Fisher’s exact test, respectively

**Fig. 1:**
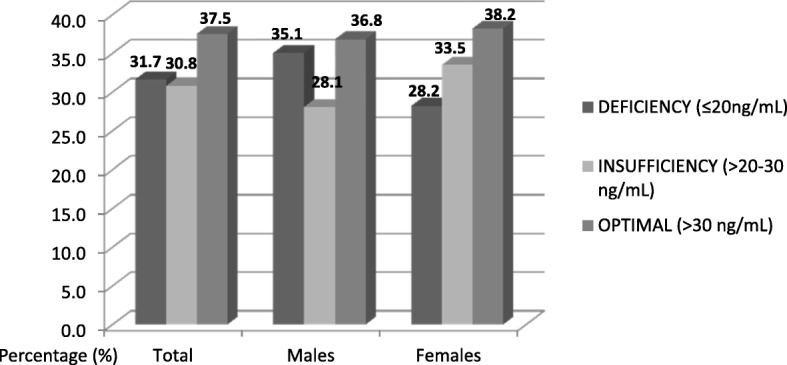
Vitamin D Status of Study Participants According to the National Osteoporosis Foundation Cutoffs [3]

**Fig. 2:**
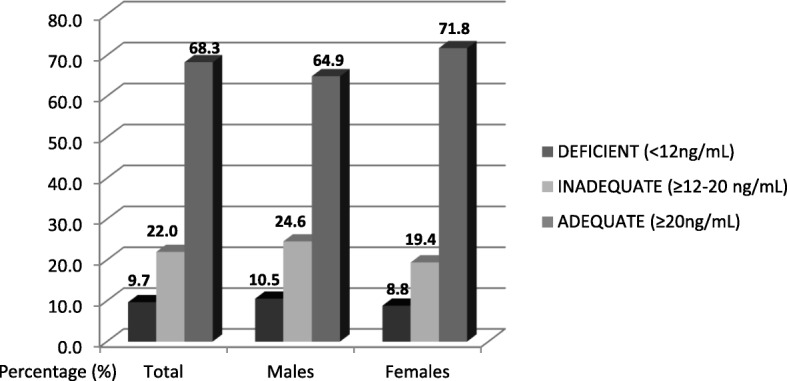
Vitamin D Status of Study Participants According to the Institute of Medicine Cutoffs [2]

In the total sample, mean vitamin D serum concentration was 28.2 ± 13.9 ng/mL. There were no significant differences between genders. Among study participants, 37.5% had serum 25(OH)D concentrations ≥30 ng/mL, 31.7% had serum 25(OH)D concentrations < 20 ng/mL. Further, 9.7% of participants had 25(OH)D concentrations < 12 ng/mL. There was no significant difference in vitamin D status between men and women (Figs. 1 and 2).

The associations of socio-demographic, dietary, lifestyle, and anthropometric factors with insufficient/sufficient vitamin D status (defined as 25(OH)D ≤ or > 30 ng/ml) were presented in Table 2. In the total sample, vitamin D intake, sun exposure, PBF, and WC were associated with vitamin D status. Individuals with sufficient vitamin D status had higher vitamin D intake (3.3 ± 5.7 μg) than in the insufficient group (1.9 ± 2.0 μg) (*p* = 0.047). A higher percentage (41.0%) of those spending > 60 mins of daily sun exposure had sufficient vitamin D status compared to those spending ≤15 mins (21.4%, *p* = 0.005). Among all participants, the mean PBF was lower in those having sufficient vitamin D status (27.2 ± 7.5%) compared to those having insufficient status (31.2 ± 7.5%, *p* < 0.005). Among individuals having high risk WC, only 20.3% had sufficient vitamin D status while 79.7% had insufficient vitamin D status (*p* = 0.012). In men, only sun exposure and PBF were associated with vitamin D status. A higher percentage (42.6%) of those spending > 60 mins of daily sun exposure had sufficient vitamin D status compared to those spending ≤15 mins (20.4%, *p* = 0.028). In men, the mean PBF was lower in those having sufficient vitamin D status (25.6 ± 7.5%) compared to those having insufficient status (28.3 ± 6.6%, *p* = 0.039). In women, vitamin D intake, alcohol intake, BMI, PBF, and WC were associated with vitamin D status. Individuals with sufficient vitamin D status (> 30 ng/mL) had higher mean vitamin D intake (3.1 ± 2.7 μg) than in the insufficient group (1.6 ± 1.7 μg) (*p* = 0.006). Among female alcohol drinkers, 45.8% had sufficient vitamin D status compared to 20.6% of female alcohol non-drinkers (*p* = 0.022). Among obese women, 95.0% had insufficient vitamin D status, while only 5.0% had sufficient vitamin D status (*p* = 0.008). In women, the mean PBF was lower in those having sufficient vitamin D status (29.3 ± 7.0%) compared to those having insufficient status (34.4 ± 7.0%, *p* = 0.001). Among women having high risk WC, 87.1% had insufficient vitamin D status, while only 12.9% had sufficient vitamin D status (*p* = 0.003).

**Table 2:** Associations of socio-demographic, dietary, lifestyle, anthropometric factors with Vitamin D status^* *^among study participants

Characteristic	Total (*n* = 268)			Men (*n* = 142)			Women (*n* = 126)		
	Insufficient	Sufficient	*P*-value^3^	Insufficient	Sufficient	*P*-value^3^	Insufficient	Sufficient	*P*-value^3^
	Mean ± SD / n (%)/ Median [IQR]			Mean ± SD / n (%)			Mean ± SD / n (%)		
Age (years)	40 [33.0–51.0	43 [35.0–51.0]	0.593	45.8 ± 12.3	42.1 ± 11.2	0.088	37.3 ± 9.8	39.3 ± 9.7	0.320
Marital status			0.140			0.042			0.976
Single/Separated/ Divorced	56 (65.9)	29 (34.1)		23 (57.5)	17 (42.5)		33 (73.3)	12 (26.7)	
Married	138 (75.4)	45 (24.6)		77 (75.5)	25 (24.5)		61 (75.3)	20 (24.7)	
Education level			0.793			0.551			0.231
High school	45 (70.3)	19 (29.7)		30 (68.2)	14 (31.8)		15 (75.0)	5 (25.0)	
Bachelor degree	52 (75.4)	17 (24.6)		20 (64.5)	11 (35.5)		32 (84.2)	6 (15.8)	
Graduate	97 (71.9)	38 (28.1)		50 (74.6)	17 (25.4)		47 (69.1)	21 (30.9)	
Income ($)			0.902			0.137			0.295
< 2250	68 (70.8)	28 (29.2)		34 (61.8)	21 (38.2)		34 (82.9)	7 (17.1)	
2250–4000	51 (73.9)	18 (26.1)		23 (82.1)	5 (17.9)		28 (68.3)	13 (31.7)	
> 4000	75 (72.8)	28 (27.2)		43 (72.9)	16 (27.1)		32 (72.7)	12 (27.3)	
Vitamin D intake (μg)	1.9 ± 2.0	3.3 ± 5.7	0.047	2.2 ± 2.2	3.4 ± 7.2	0.290	1.6 ± 1.7	3.1 ± 2.7	0.006
Alcohol drinking			0.095			0.964			0.022
No	145 (75.5)	47 (24.5)		64 (71.1)	26 (28.9)		81 (79.4)	21 (20.6)	
Yes	49 (64.5)	27 (35.5)		36 (69.2)	16 (30.8)		13 (54.2)	11 (45.8)	
Smoking			0.563			1			0.480
No	122 (73.9)	43 (26.1)		55 (70.5)	23 (29.5)		67 (77.0)	20 (23.0)	
Yes	72 (69.9)	31 (30.1)		45 (70.3)	19 (29.7)		27 (69.2)	12 (30.8)	
Daily exposure to direct sunlight			0.005			0.028			0.206
≤ 15 mins	81 (78.6)^a^	22 (21.4)^a^		43 (79.6)^a^	11 (20.4)^a^		38 (77.6)	11 (22.4)	
16–60 min	64 (78.0)^a^	18 (22.0)^a^		26 (76.5)^a,b^	8 (23.5)^a,b^		38 (79.2)	10 (20.8)	
> 60 mins	49 (59.0)^b^	34(41.0)^b^		31 (57.4)^b^	23 (42.6)^b^		18 (62.1)	11 (37.9)	
Use sunscreen			0.744			0.670			0.185
No	142 (73.2)	52 (26.8)		95 (69.9)	41 (30.1)		47 (81.0)	11 (19.0)	
Yes	52 (70.3)	22 (29.7)		5 (83.3)	1 (16.7)		47 (69.1)	21 (30.9)	
BMI^1^ (Kg/m^2^)			0.242			0.479			0.008
Underweight	0 (0.0)	0 (0.0)		0 (0.0)	0 (0.0)		0 (0.0)	0 (0.0)	
Normal	68 (68.0)	32 (32.0)		20 (76.9)	6 (23.1)		48 (64.9)^b^	26 (35.1)^a^	
Overweight	71 (71.7)	28 (28.3)		44 (65.7)	23 (34.3)		27 (84.4)^a^	5 (15.6)^a^	
Obese	55 (79.7)	14 (20.3)		36 (73.5)	13 (26.5)		19 (95.0)^b^	1 (5.0)^a^	
Percent body fat	31.2 ± 7.5	27.2 ± 7.5	0.000	28.3 ± 6.6	25.6 ± 7.5	0.039	34.4 ± 7.0	29.3 ± 7.0	0.001
Waist circumference risky			0.012			0.581			0.003
No	88 (65.2)	47 (34.8)		48 (67.6)	23 (32.4)		40 (62.5)	24 (37.5)	
Yes^2^	106 (79.7)	27 (20.3)		52 (73.2)	19 (26.8)		54 (87.1)	8 (12.9)	

^*^The National Osteoporosis Foundation cutoffs were used to define vitamin D status (Sufficient: 25hydroxyvitamin D > 30 ng/mL and Insufficient: 25 hydroxyvitamin D ≤ 30 ng/mL)

^*^Participants taking vitamin D supplements were excluded (*n* = 75)

^1^Body Mass Index

^2^ < 88 cm for women and < 102 cm for men [24]

^3^The P value reflects differences in vitamin D status

Columns with superscripts without a common symbol differ, the *P* value is < 0.05

Comparisons of continuous and categorical variables were performed using independent sample T Test/Mann-Whitney-U-test and the chi square test /Fisher’s exact test, respectively

Socio-demographic, dietary, lifestyle, and anthropometric factors were not associated with adequate/inadequate vitamin D status (defined as 25(OH)D < or ≥ 20 ng/ml) (Data not shown).

Using multiple logistic regression analysis to examine the association between body composition and insufficient vitamin D status (defined as 25(OHD ≤ 30 ng/mL), it was found that for each 1% increase in body fat, participants were around 8% more likely to have insufficient vitamin D status (*p* = 0.019) while controlling for BMI, age, gender, education, sunscreen use, vitamin D intake, intake of vitamin D supplements, medical condition, sunlight exposure, and intake of OCPs (Table 3). Using multiple logistic regression analysis to examine the association between body composition and inadequate vitamin D status (defined as 25(OHD <  20 ng/mL), it was shown that for each 1% increase in body fat, participants were around 6% more likely to have inadequate vitamin D status while controlling for the same factors in addition to alcohol intake and hypertriglyceridemia. However, the association was found to be of borderline significance (*p* = 0.054) (Table 4).

**Table 3:** Multivariable logistic regression for body composition and Vitamin D Status (≤30 ng/mL) among study participants^*^

Characteristic	Odds Ratio (OR)	95% C.I		*P* value
		Lower	Upper	
Percent Body Fat	1.077	1.012	1.145	0.019
BMI^1^ (kg/m^2^)	0.969	0.881	1.066	0.521
Age	0.976	0.950	1.004	0.092
Gender	0.568	0.227	1.419	0.226
Education				
Bachelor degree * High School	1.007	0.455	2.229	0.986
Graduate ^*^ High School	1.059	0.523	2.145	0.874
Sunscreen Use	0.827	0.415	1.651	0.591
Chronic Disease	1.478	0.799	2.735	0.213
Vitamin D intake (μg)	0.837	0.746	0.941	0.003
Sun Exposure				
16–60 min * ≤15 mins	1.271	0.670	2.413	0.463
> 60 mins * ≤15 mins	0.485	0.258	0.912	0.025
Use of OCP^2^	0.346	0.019	6.154	0.470
Vitamin D supplements	0.084	0.042	0.169	0.000

^*^The National Osteoporosis Foundation cutoffs were used to define vitamin D status (Sufficient: 25hydroxyvitamin D > 30 ng/mL and Insufficient: 25 hydroxyvitamin D ≤ 30 ng/mL)

^1^Body Mass Index

^2^Oral Contraceptive Pills

**Table 4:** Multivariable logistic regression for body composition and Vitamin D status (< 20 ng/mL) among study participants*

Characteristic	Odds Ratio (OR)	95% C.I		*P* value
		Lower	Upper	
Percent Body Fat	1.062	0.999	1.130	0.054
BMI^1^ (kg/m^2^)	0.935	0.854	1.024	0.148
Age	0.972	0.944	1.000	0.049
Gender	0.466	0.188	1.157	0.1
Education				
Bachelor degree * High School	0.951	0.442	2.045	0.897
Graduate * High School	1.113	0.559	2.214	0.761
Sunscreen Use	0.578	0.286	1.167	0.126
Chronic Disease	1.036	0.552	1.941	0.913
Vitamin D Intake (μg)	0.829	0.717	0.958	0.011
Sun Exposure				
16–60 min * ≤ 15 mins	0.914	0.492	1.698	0.776
> 60 mins * ≤ 15 mins	0.747	0.396	1.412	0.370
Use of OCP^2^	2.140	0.122	37.443	0.602
Vitamin D Supplements	0.131	0.052	0.333	0.000
Alcohol Intake	0.422	0.222	0.801	0.008
Hypertriglyceridemia^3^	2.189	1.153	4.157	0.017

^*^The Institute of Medicine cutoffs were used to define vitamin D status (Adequate: 25hydroxyvitamin D ≥ 20 ng/mL and Inadequate: 25 hydroxyvitamin D < 20 ng/mL)

^1^Body Mass Index

^2^Oral Contraceptive Pills

^3^ ≥ 150 mg/dL [48]

## Discussion

The current study showed that low vitamin D status was prevalent in Lebanese adults during fall, with more than 30 and 60% of participants having serum 25(OH)D concentrations < 20 ng/mL and ≤ 30 ng/mL, respectively. BMI was not associated with vitamin D status; however PBF was associated with insufficient vitamin D status using the NOF cutoffs.

Low vitamin D status has become a major problem worldwide, even in sunny countries like Lebanon [27]. The prevalence of low vitamin D status reported in our study (60%) was in line with the prevalence rates reported in neighboring countries ranging from 57.6% in Tehran [28] to up to 91% in Morocco [29]. The range of prevalence rates varied among studies due to the different study populations, education levels, season, BMI, age, body composition, gender, and cutoffs for vitamin D status. Our results were concordant with the prevalence rate of 73.3% among 105 Lebanese adults (aged > 18 years) visiting outpatient clinics between April and July reported by Rachkidi and Aoun, 2015 [6]. On the other hand, Gannage et al. 2014, reported a higher prevalence of 94.9% among employees in a university hospital (*n* = 329) [11]. In the latter study, data was collected over a year; accordingly, some participants were recruited in the winter season, which could increase the likelihood of low vitamin D status. The seasonal variation in vitamin D concentration has been previously reported in the literature [30]. Hoteit et al., 2014 reported that 25(OH)D concentrations were lower in the winter season (20.1 ± 9.2 ng/mL) among 9147 Lebanese outpatients compared to 24.8 ± 10.4 ng/mL in the fall season [5]. The mean serum 25(OH)D concentration reported in our study was 28.2 ± 13.9 ng/mL, which was similar to the mean reported by Hoteit et al., 2014 during the same season.

In our study, in bivariate analyses, longer duration of sun exposure was associated with better vitamin D status in the total sample and in men but not in women. Due to highly pigmented skin, individuals of Middle Eastern origin might need to spend more time in the sun to synthesize sufficient amounts of vitamin D endogenously [31]. This justification is backed up by our multivariate logistic analyses; since sun exposure > 1 h was protective against low vitamin D status, while sun exposure between 16 and 60 min was not protective. It is likely we did not observe this association in women in bivariate analyses, since around 54% of women were using sunscreen, while only 4% of men were using sunscreen. It is well established in the literature that sunscreen use can block UVB light and reduce vitamin D synthesis significantly [32]. The association between sun exposure and vitamin D status is inconsistent in the literature, since several factors may affect the cutaneous synthesis and bioavailability of vitamin D, such as the time of day, the use of sunscreen, and skin pigmentation [6, 32, 33]. These factors could explain gender differences in our sample.

In bivariate analyses, vitamin D intake was associated with vitamin D status in the total sample, and in women but not in men. Women who had a sufficient status had a higher vitamin D intake compared to women who had an insufficient status; this same trend was observed in men but did not reach statistical significance. Vitamin D is obtained from two sources, diet and sun exposure [1]. While sun exposure was not associated with vitamin D status in women due to the use of sunscreen, diet seems to be the most important factor affecting vitamin D status for women; however, the sun was the most important factor affecting vitamin D status for men [32]. However, in multivariate logistic regression, once all confounders were controlled for, sun exposure, vitamin D intake, and vitamin D supplements became protective against low status, which is concordant with the literature [6, 25, 34].

Mean vitamin D intake (2.2 ± 3.2 μg/day) reported in our study was similar to that reported by Rachkidi and Aoun, 2015 (2.05 ± 1.69 μg/day). Both studies show low intakes of vitamin D compared to the DRI of 15 μg/day [35]. It is important to point out that vitamin D is found only in a few foods. Furthermore, the fortification of milk in Lebanon is not mandatory compared to other countries, such as the US and Canada [36]. In addition, the consumption of milk was low in our sample (0.97 ± 1.3 servings/day) which is below the recommendations set by the Lebanese Food-Based Dietary Guidelines of 3 cups/day [37]. Old evidence suggested a high prevalence of lactose intolerance in the Lebanese population; however, new evidence is lacking [38].

The association between alcohol intake and vitamin D status was observed in women only; this has been previously reported in the literature [39–41]. In our sample, women who drank alcohol had a higher prevalence of sufficient vitamin D status compared to non-drinkers. The relationship between alcohol intake and vitamin D status is not well understood, and results are still inconclusive. It is likely that this relationship is affected by confounders; especially that alcohol intake was not associated with vitamin D status in multivariable analyses.

Measures of adiposity, including WC and PBF, were inversely associated with vitamin D status in all participants and in both genders, while BMI was inversely associated with vitamin D status in women only. The association between BMI and vitamin D status is not always consistent in the literature [25], since BMI has many limitations, as it does not necessarily reflect the PBF [42]. The association of adiposity, measured by WC and PBF, with serum 25(OH)D is usually stronger than that with BMI [43]. In concordance with our results, multiple studies showed that vitamin D concentrations were significantly lower in both males and females with high risk WC compared to low risk categories (*p* < 0.05) [9, 11, 17].

Our study found a positive association between low vitamin D status (25(OH)D ≤ 30 ng/mL) and PBF, which is also concordant with the literature [17, 25, 34, 44]. While this relationship is not fully understood, it is assumed that since vitamin D is a fat soluble vitamin, it might be sequestered in the excess adipose tissue of obese adults and would be slowly released at negative energy balance [17]. Further, a potential confounder is that obesity is also linked to an unhealthy lifestyle, characterized by less physical activity, less sun exposure and, hence, lower vitamin D concentrations [45].

Using the IOM cutoffs to define vitamin D status as inadequate (25(OH)D < 20 ng/mL) the association between vitamin D status and PBF was observed but did not reach statistical significance (*p* > 0.05). It is possible that the association between vitamin D status and body composition is not observed unless vitamin D concentrations are high, since NOF cutoffs are higher than IOM cutoffs [2, 3].

The present study has some limitations that need to be acknowledged. First, the study design was cross-sectional, which does not allow drawing causal relationships between vitamin D status and measures of adiposity [46]. Second, the study was performed on employees from a private university in Lebanon, which limits generalizability to the Lebanese population. In addition, BIA was used for the assessment of body composition. The dual-energy x-ray absorptiometry (DEXA), computed tomography (CT), and magnetic resonance imaging (MRI) are considered the gold standard imaging modality for the precise estimation of amount of adipose tissue in various body regions [16]. However, DEXA, CT and MRI imaging are impractical for screening the general population, since they are expensive and invasive [34]. For the assessment of vitamin D concentration, ELISA, the most commonly used technique, was used, since it is simple and inexpensive, yet it is less reliable than the gold standard technique, HPLC. HPLC is not commonly used because of its complexity and limitations including the need for specialized staff, the large volume of blood required, and the longer turnaround time [47]. Data was collected between mid-October to mid-December, which means that we cannot compare our results to studies performed in different seasons. Vitamin D content of many foods listed in the FFQ were not available in the Lebanese food composition tables; instead, the Canadian Nutrient File was used to estimate the vitamin D content of these foods.

To our knowledge, it is the first study in Lebanon to assess the association between body composition and vitamin D status, while controlling for BMI and other important confounders [18].

## Conclusion

Our results support our hypothesis confirming that PBF is positively associated with low vitamin D status independent of BMI in our sample of university employees. Accordingly, education about the importance of consuming high sources of vitamin D is primordial, particularly since living in a sunny country might undermine the need to focus on diet, as Lebanese might believe that sun exposure is sufficient to maintain healthy vitamin D status. Further, this study reinforces the need for regular screening for low vitamin D status in Lebanese adults, particularly among individuals at risk, including those with high risk WC, high PBF, who work indoors, and have low vitamin D intake, and recommending vitamin D supplementation if needed. As low vitamin D status has been recently associated with many chronic diseases, a nationwide assessment of vitamin D status is required among different age and gender groups across different seasons to identify whether the government needs to consider the fortification of milk at the national level.

Moreover, cohort studies examining the association between body fat and vitamin D are needed to address the temporal relationship with vitamin D status.

## Abbreviations

AGS: American Geriatrics Society
BIA: Bioelectrical impedance analysis
BMI: Body mass index
CT: Computed tomography
DEXA: Dual-energy X-ray absorptiometry
ELISA: Enzyme linked immunosorbent assay
FFQ: Food frequency questionnaire
IOM: Institute of Medicine
IPAQ: International physical activity questionnaire
LDL: Low density lipoprotein
MRI: Magnetic resonance imaging
NDU: Notre Dame University
NOF: National Osteoporosis Foundation
OCP: Oral contraceptive pills
PBF: Percent body fat
SPSS: Statistical Package for Social Sciences
WC: Waist circumference
WHO: World Health Organization

## References

[1] Chen TC, Chimeh F, Lu Z, Mathieu J, Person KS, Zhang A (2007). Factors that influence the cutaneous synthesis and dietary sources of vitamin D. Arch Biochem Biophys.

[2] Ross AC, Manson JE, Abrams SA, Aloia JF, Brannon PM, Clinton SK (2011). The 2011 report on dietary reference intakes for calcium and vitamin D from the institute of medicine: what clinicians need to know. J Clin Endocrinol Metab.

[3] Holick MF, Binkley NC, Bischoff-Ferrari HA, Gordon CM, Hanley DA, Heaney RP (2011). Evaluation, treatment, and prevention of vitamin D deficiency: an endocrine society clinical practice guideline. J Clin Endocrinol Metab.

[4] Mithal A, Wahl DA, Bonjour JP, Burckhardt P, Dawson-Hughes B, Eisman JA (2009). Global vitamin D status and determinants of hypovitaminosis D. Osteoporos Int.

[5] Hoteit M, Al-Shaar L, Yazbeck C, Sleiman MB, Ghalayini T, Fuleihan GE (2014). Hypovitaminosis D in a sunny country: time trends, predictors, and implications for practice guidelines. Metabolism.

[6] Rachkidi D, Aoun A (2014). Vitamin D status and determinants among ambulatory patients: a cross-sectional study. Clin Nutr.

[7] Florez H, Martinez R, Chacra W, Strickman-Stein N, Levis S (2007). Outdoor exercise reduces the risk of hypovitaminosis D in the obese. J Steroid Biochem Mol Biol.

[8] Vanlint S (2013). Vitamin D and obesity. Nutrients.

[9] Dahbani K, Tsulidis KK, Murphy N, Ward HA, Bliott P, Riboli E, et al. Prevalence of vitamin D deficiency and association with metabolic syndrome in Qatari population. Nutr Diabetes. 2017;7:e263. 10.1038/nutd.2017.14 PMC5436094 28394362

[10] Sadiya A, Ahmed A, Skaria S, Abusnana S. Vitamin D status and its relationship with metabolic markers in persons with obesity and type 2 diabetes in the UAE: a cross-sectional study. J Diabetes Res. 2014;2014. 10.1155/2014/869307. 10.1155/2014/869307 PMC4211253 25371907

[11] Gannagé-Yared MH, Helou E, Zaraket V (2014). Serum 25 hydroxyviamin D in employees of a middle eastern university hospital. J Endocrinol Investig.

[12] Fuleihan G (2009). Vitamin D deficiency in the Middle East and its health consequences. Holick MF vitamin D: physiology, molecular biology, and clinical applications.

[13] Golbahar J, Al-Saffar N, Altayab Diab D, Al-Othman S, Darwish A, Al-Kafaji G (2014). Predictors of vitamin D deficiency and insufficiency in adult Bahrainis: a crosssectional study. Public Health Nutr.

[14] Gannage-Yared MH, Chedid R, Khalife S, Azzi E, Zoghbi F, Halaby G (2009). Vitamin D in relation to metabolic risk factors, insulin sensitivity and adiponectin in a young middle-eastern population. Eur J Endocrinol.

[15] Andreozzi P, Verrusio W, Viscogliosi G, Summa ML, Gueli N, Cacciafesta M (2016). Relationship between vitamin D and body fat distribution evaluated by DXA in postmenopausal women. Nutrition.

[16] Kim D, Kim J (2016). Association between serum 25-hydroxyvitamin D levels and adiposity measurements in the general korean population. Nutr Res Pract.

[17] González L, Ramos-Trautmann G, Díaz-Luquis GM, Pérez CM, Palacios C (2015). Vitamin D status is inversely associated with obesity in a clinic-based sample in Puerto Rico. Nutr Res.

[18] Bassil D, Rahme M, Hoteit M, Fuleihan G (2013). Hypovitaminosis D in the Middle East and North Africa: prevalence, risk factors and impact on outcomes. Dermatoendocrinol.

[19] Booth M (2000). Assessment of physical activity: an international perspective. Res Q Exerc Sport.

[20] El Hayek J, Pham T, Finch S, Hazell T, Vanstone C, Weiler H (2014). Validity and reproducibility of a short food frequency questionnaire in assessing calcium and vitamin D intake in Canadian preschoolers. EC Nutr.

[21] Block G, Coyle LM, Hartman AM, Scoppa SM (1994). Revision of dietary analysis software for the health habits and history questionnaire. Am J Clin Nutr.

[22] Pellet P, Shadarevian S (1970). Food composition tables for use in the middle east.

[23] Government of Canada. Canadian nutrient file 2016. https://food-nutrition.canada.ca/cnf-fce/index-eng.jsp. Accessed Jan 2018.

[24] WHO (2008). Waist circumference and waist-hip ratio. Report of a WHO expert consultation.

[25] Shantavasinkul PC, Phanachet P, Puchaiwattananon O, Chailurkit L, Lepananon T, Chanprasertyotin S (2015). Vitamin D status is a determinant of skeletal muscle mass in obesity according to body fat percentage. Nutrition.

[26] Baracos V, Caserotti P, Earthman CP, Fields D, Gallagher D, Hall KD (2012). Advances in the science and application of body composition measurement. JPEN.

[27] Gannagé-Yared MH, Chemali R, Yaacoub N, Halaby G (2000). Hypovitaminosis D in a sunny country: relation to lifestyle and bone markers. JBMR.

[28] Hashemipour S, Larijani B, Adibi H, Javadi E, Sedaghat M, Pajouhi M (2004). Vitamin D deficiency and causative factors in the population of Tehran. BMC Public Health.

[29] Allali F, El Aichaoui S, Khazani H, Benyahia B, Saoud B, El Kabbaj S (2009). High prevalence of hypovitaminosis D in Morocco: relationship to lifestyle, physical performance, bone markers, and bone mineral density. Semin Arthritis Rheum.

[30] Bolland MJ, Grey AB, Ames RW, Mason BH, Horne AM, Gamble GD (2007). The effects of seasonal variation of 25-hydroxyvitamin D and fat mass on a diagnosis of vitamin D sufficiency. Am J Clin Nutr.

[31] Hagenau T, Vest R, Gissel T, Poulsen C, Erlandsen M, Mosekilde L (2009). Global vitamin D levels in relation to age, gender, skin pigmentation and latitude: an ecologic meta-regression analysis. Osteoporos Int.

[32] Matsuoka LY, Wortsman J, Hollis BW (1990). Use of topical sunscreen for the evaluation of regional synthesis of vitamin D3. J Am Acad Dermatol.

[33] Harris SS, Dawson-Hughes B (2007). Reduced sun exposure does not explain the inverse association of 25-hydroxyvitamin D with percent body fat in older adults. J Clin Endocrinol Metab.

[34] Savastano S, Barrea L, Savanelli MC, Nappi F, Di Somma C, Orio F (2017). Low vitamin D status and obesity: role of nutritionist. Rev Endocr Metab Disord.

[35] Institute of Medicine (2011). Dietary reference intakes for calcium and vitamin D.

[36] Calvo MS, Whiting SJ, Barton CN (2004). Vitamin D fortification in the United States and Canada: current status and data needs. Am J Clin Nutr.

[37] Hwalla N, Nasreddine L, Jarjar AF (2013). The food-based dietary guideline manual for promoting healthy eating in the Lebanese adult population. The American University of Beirut.

[38] Nasrallah SM (1979). Lactose intolerance in the lebanese population and in mediterranean lymphoma. Am J Clin Nutr.

[39] Jacques PF, Felson DT, Tucker KL, Mahnken B, Wilson PW, Rosenberg IH (1997). Plasma 25-hydroxyvitamin D and its determinants in an elderly population sample. Am J Clin Nutr.

[40] Lee K (2012). Sex-specific relationships between alcohol consumption and vitamin D levels: the Korea national health and nutrition examination survey 2009. Nutr Res Pract..

[41] McCullough ML, Weinstein SJ, Freedman DM, Helzlsouer K, Flanders WD, Koenig K (2010). Correlates of circulating 25-hydroxyvitamin D: cohort consortium vitamin D pooling project of rarer cancers. Am J Epidemiol.

[42] Ode JJ, Pivarnik JM, Reeves MJ, Knous JL (2007). Body mass index as a predictor of percent fat in college athletes and nonathletes. Med Sci Sports Exerc.

[43] Arunabh S, Pollack S, Yeh J, Aloia JF (2003). Body fat content and 25-hydroxyvitamin D levels in healthy women. J Clin Endocrinol Metab.

[44] Parikh SJ, Edelman M, Uwaifo GI, Freedman RJ, Semega-Janneh M, Reynolds J (2004). The relationship between obesity and serum 1, 25-dihydroxy vitamin D concentrations in healthy adults. J Clin Endocrinol Metab.

[45] Osei K (2010). 25-OH vitamin D: is it the universal panacea for metabolic syndrome and type 2 diabetes?. J Clin Endocrinol Metab.

[46] Aasheim ET, Hofso D, Hjelmesaeth J, Birkeland KI, Bohmer T (2008). Vitamin status in morbidly obese patients: a cross-sectional study. Am J Clin Nutr.

[47] Wallace AM, Gibson S, de la Hunty A, Lamberg-Allardt C, Ashwell M (2010). Measurement of 25-hydroxyvitamin D in the clinical laboratory: current procedures, performance characteristics and limitations. Steroids.

[48] Huang PL. A comprehensive definition of metabolic syndrome. Dis Model Mech. 2009;2(5–6):231–7.. 10.1242/dmm.001180 PMC2675814 19407331

